# Association of siblings’ presence and oral health-related quality of life among children: a cross-sectional study

**DOI:** 10.1186/s12903-021-01526-y

**Published:** 2021-03-24

**Authors:** Min Liu, Qingping Yun, Mei Zhao, Wei Chen, Hui Zhang, Wei Hou, Chun Chang

**Affiliations:** 1 Department of Preventive Dentistry, Beijing Stomatological Hospital, Capital Medical University, Beijing, China; 2 Department of Social Medicine and Health Education, School of Public Health, Peking University, No.38 Xueyuan Road, Haidian District, Beijing, 100191 China

**Keywords:** Oral Health-Related Quality of Life, Sibling, Interaction effect, Schoolchildren

## Abstract

**Background:**

The quantity–quality trade-off theory indicates that an increase in siblings might decrease a child’s well-being, but little is known about the impacts of sibling number on children’s oral health-related quality of life (OHRQoL). This study aims to investigate the effects of presence of siblings on children’s OHRQoL, and to further test whether there is an interaction effect between siblings’ presence and locations on children’s OHRQoL.

**Methods:**

Data were obtained from an oral-health survey of 12-year-old children in Beijing, China, which was a part of the 4th National Oral Health Survey in the Mainland of China (2015–2016). This study included 2158 children data for analysis. OHRQoL was assessed by the child’s Oral Impacts on Daily Performance (OIDP). OIDP prevalence served as an indicator of OHRQoL. Children with and without siblings were recorded as non-single and single children, respectively. Dental variables, including active caries, gingival bleeding, and calculus, were reported. A logistic regression model was applied to investigate the association of siblings’ presence and OIDP prevalence. A synergy index was used to assess the possible interaction effect between siblings’ presence and location on OIDP prevalence.

**Results:**

Sixty percent of Chinese children reported suffering OIDP in the previous six months. OIDP prevalence for non-single and single children were 68.3% and 56.9%, respectively. The adjusted odds ratio (OR) of OIDP for non-single children was 1.31 (95% CI: 1.05, 1.63), and the adjusted OR of OIDP for non-single and rural children was 2.03 (95% CI: 1.47, 2.81). The synergy index between siblings’ presence and location on OIDP was 2.18 (85% CI: 1.30, 3.67), which indicates that an excessive risk increase for OIDP was observed among non-single and rural children.

**Conclusions:**

Children with siblings are more likely to report OIDP and have lower OHRQoL, especially those from rural areas. These findings indicate that oral-health interventions should be given priority for non-single and rural children.

**Supplementary Information:**

The online version contains supplementary material available at 10.1186/s12903-021-01526-y.

## Background

The Global Burden of Disease Study 2017 estimated that oral diseases affect nearly 3.5 billion people worldwide [1]. It is estimated that 60–90% of school‐aged children globally are affected by oral health problems [2], and these problems negatively affect child overall health [3]. According to the World Dental Federation, oral health is multi-faceted and includes the ability to speak, smile, smell, taste, touch, chew, swallow, and convey a range of emotions through facial expressions with confidence and without pain, discomfort, and disease of the craniofacial complex [4]. In this broader concept of oral health, assessment of oral health is not only focused on the biological level, but also on emotional and social functional level.

Oral health-related quality of life (OHRQoL) is a self-report measurement of oral health status, which captures functional, social, and psychological impacts of oral disease [5]. Unlike traditional measurements focusing on clinical indices, OHRQoL is more concerned about impact of oral health problems on quality of life [6]. As a generic instrument, OHRQoL can investigate the impact of oral health conditions in relation to general health perceptions, allowing for comparisons between different diseases [7]. More importantly, OHRQoL can simplify understanding of health burdens of oral disease for health policy makers [8]. As oral health is crucial for general health, OHRQoL is recognized by World Health Organization (WHO) and serves as a segment of the Global Oral Health Program [9].

OHRQoL is not only affected by oral health conditions, but also related to socio-demographic and contextual factors [10]. Previous studies showed that children with different demographical backgrounds [11] (e.g., gender, ethnic background) and family socioeconomical status [12] had different OHRQoL. The siblings’ number is also a key family characteristic. Previous studies found siblings’ number was negatively associated with child health [13, 14] and health expenditure [15]. This negative association is consistent with quantity–quality trade-off perspective. According to quantity-quality trade-off theory, an increase in the quantity of children tends to decrease available resources for investing in the human capital per child, which consequently leads to a trade-off between quantity and quality of children [27]. However, little is known about the relationship between number of sibling and children’s OHRQoL.

Although evidence of association between sibling’s number and OHRQoL is lacking, there are some evidences linking sibling’s number with other oral health outcomes. Having two or more siblings is associated with lower odds of regular brushing and annual dental visits [16], and having siblings is more likely to increase the incidence of caries [17]. In particular, caries are more common in the second born child than the first child [18], further supporting this negative relationship. These existing evidences show that the sibling number is negatively associated with dental visits as well as oral health condition. Moreover, dental caries was a common oral condition affecting children’s OHRQoL [19], and dental visit was also associated with OHRQoL [20]. Current evidences suggest a negative association between sibling’s number and children’s OHRQoL.

Thus, the aim of the current study is to examine the association between presence of siblings and children’s OHRQoL by using 12-year-old children oral health survey data in Beijing, China. Considering the significant urban–rural disparities in socioeconomic situations and healthcare accessibility in China [21], and both socioeconomic status and dental-care attendance are positively associated with OHRQoL [22], there is a need to further access the possible interaction effect between siblings’ presence and locations on children’s OHRQoL. There is also a need to see whether the combined demographical characteristics will result in a greater effect on children’s OHRQoL.

## Methods

### Data resource

This survey was a part of the 4th National Oral Health Survey in the Mainland of China (2015–2016), and it was designed to provide provincial-level estimates [23]. The key variable to calculate sample size was caries prevalence. The sample size was estimated by using the formula $N=\mathrm{Deff}\frac{{{U}_{\alpha }}^{2}(1-\mathrm{P})\mathrm{P}}{{\mathrm{d}}^{2}}$ with parameters as listed below: (1) the prevalence (P) of caries among 12-year-old children in Beijing in the 3rd oral health survey in 2005 was 28.9%; (2) the level of significance was at 5%; (3) the deviation (d) = 0.1P; (4) design effect (Deff) = 2. Considering the non-response rate was 10%, the sample size for the survey was 2079. The multistage cluster sampling was used in this survey. First, 6 out of 18 districts in Beijing were sampled using a digital random table. Second, 3 schools were sampled from each district using the probability proportional to size sampling method. Third, 60 male and 60 female schoolchildren were enrolled in each selected school using the cluster sampling method. The recruitment started from the first class in 7th grade; additional children were selected from the next class. In total, 2160 children were enrolled. All children participated in both clinical dental examinations and questionnaire surveys. Two students failed to finish the questionnaire and were excluded from the data analysis. Therefore, the data from 2158 children were used in this analysis. According to the prevalence of oral impact daily performance (OIDP) reported among Chinese schoolchildren in Urumqi in 2008 [24], a sample of 1499 schoolchildren would be necessary for the current analysis using the above parameters. Thus, 2158 children met the study’s sample size requirement. 

### Measurement of OHRQoL

OHRQoL was measured using the eight-item Child Oral Impacts on Daily Performance (OIDP). The Child-OIDP was initially developed in English in Thailand [25], and was shown to be valid and reliable when applied to Chinese schoolchildren [24]. The children were asked about the impacts of their oral health condition on their daily life. The eight items included “has your oral health status affected eating/ speaking/ mouth cleaning/ sleeping/ emotion/ smiling/ studying/ social contact in the past six months?” The severity of impact is as follows: no impact, little impact, moderate impact, and severe impact. The results of an internal reliability analysis of the Child-OIDP in the current sample showed that there were positive correlations between items, and the correlation coefficients were between 0.34 and 0.61. The Cronbach’s alpha coefficient was 0.85 (see Additional file 1). Each item was dichotomized as with impact (little/moderate/severe impact, scored as 1) and without impact (no impact, scored as 0). The Child-OIDP simple count (SC) score (range 0–8) was constructed by summing 8 items. OIDPSC score (0–8) was dichotomized as 0/1+, producing the categories “no daily performance affected” and “having daily performance affected” [26, 27].

### Measurement of oral health problems

Oral examinations were conducted in accordance with the oral health survey methods recommended by the WHO, and all teeth were examined in a systematic order using the Federation Dentaries International tooth numbering system [28]. Three examiners conducted the oral examination for children. The oral examiners should be the dental practitioners who have been engaged in oral clinical work for more than three years, and all the examiners were trained before survey. The test–retest method was used to determine the reliability of caries measurement. In the practice phase, the dentists examined 10 children, and discussed clinical diagnosis and criteria to reach the acceptable consistent level (kappa > 0.80). During the survey, 5% children were re-examined to determine the inter-examiner reliability, and the kappa score was 0.88. Portable dental chairs were carried to the classroom, and the participants were examined in a supine position. Dental caries was primarily assessed by visual inspection and then confirmed by tactile inspection using a WHO CPI probe. Although it is a full mouth examination, only the coronal part of teeth was examined. Considering that active caries were more associated with OHRQoL than caries experience, and collinearity might exist between two variables [27], active caries was reported in the current study. Active caries refers to decayed teeth (DT) diagnosed and was recorded as absence (DT = 0) or presence (DT > 0). For each tooth, the presence of gingival bleeding and calculus was evaluated using a dichotomous index, scored as 0 or 1, corresponding to absence or presence, respectively. The child was labeled as “presence of gingival bleeding” or “presence of calculus” when at least one tooth had gingival bleeding or calculus.

### Demographic characteristics

The social demographical variables in this study include gender, location, the presence of siblings, and maternal education. In China, each person is assigned a household registration type (rural/urban) based on place of birth. In the current study, child location was distinguished by the individual household registration type. The one-child policy has been implemented since 1979 and caused a discontinuity in the number of siblings. Thus, only data of whether children have siblings were collected in current study. Children with and without siblings were recorded as non-single and single children, respectively. Maternal education was recorded as junior middle school or below, high school, college, and university or above.

Demographic data and OHRQoL data were collected by a self-completed questionnaire. Teachers and interviewers co-organized and illustrated the content of the questionnaire, and the children completed questionnaire by themselves in the classroom. 

### Missing data treatment

There were 290 missing data in maternal education variables in this survey. Except for bleeding indicators, there were not statistically significant in demographic characteristics and oral health conditions between missing and non-missing data group (Additional file 2: Table 2). Therefore, the missing data might be randomly distributed. The missing data in maternal education were first filled by the father’s education, since our data showed that 59.5% of parents had same level education. If both the educations of mother and father were missing, it would be filled by exact matching. In this case, the missing data would be filled by the maternal education of another child, whose rural–urban status, sibling status and dental caries status were all the same with the child to be filled. Sensitivity analysis showed that the results of current study would not be changed before and after data imputation (Additional file 2: Table 3).

### Statistical analysis

Prevalence of each daily life impact, OIDP simple score, OIDP prevalence (OIDP score > 0), and their 95% confidence interval (CI) were calculated. A chi-square analysis and a Mann–Whitney U test were used to test the difference in OIDP scores and their prevalence, respectively. To assess effects of socio-demographic characteristics and oral health problems on a child’s OIDP, logistic regression was applied, odds ratios (OR) and their 95% CI were reported. SPSS 23.0 was used for the analyses. The biological interaction effect between presence of siblings and locations on OIDP prevalence was investigated by creating the following four (2 × 2) sub-groups: single and urban children (the reference group); single and rural children; non-single and urban children; and non-single and rural children. The examinations were conducted to see whether the effects of the three non-reference groups on a children’s OIDP were consistent with the results under the above non-interaction model. Then, the quantitative synergistic interaction was evaluated using the Synergy Index (SI) [29]:

### $SI=\frac{OR\left(AB\right)-1}{\left[OR\left(Ab\right)-1\right]+ [OR\left(aB\right)-1]}$,

where the OR is the odds ratio, Ab is exposed to one factor, aB is exposed to the other factor, and AB exposed to both factors. If the value of SI exceeds 1.0 (SI > 1), a synergistic interaction effect exists. The confidence interval (CI) of SI was estimated with this method [30]. An asymptotic covariance matrix generated by logistic regression model was used to calculate the standard error of synergy index, and an Excel sheet was used to calculate the SI and its confidence intervals (CI) [29]. As a type II error is common in interaction text [31, 32], both 95% CIs and 85% CIs of SI were calculated to avoid type II error.

All methods were carried out in accordance with relevant guidelines and regulations. Based on the male to female ratio among Beijing schoolchildren in 2015, additional analyses were performed using gender-weighted data [33]. Since results from the weighted data did not change current results (Additional file 3), we reported the results from the original data.

## Results

### OIDP score and prevalence

Table 1 shows the distribution of participants’ demographic characteristics, oral health problems, OIDP score, and OIDP prevalence. The sample of 2158 participants included 50.0% boys (*n* = 1079), 73.0% single children (*n* = 1575), and 76.1% urban children (*n* = 1643). The OIDP score of schoolchildren was 1.76 ± 2.11, and the OIDP prevalence was 60.0% (95%CI: 57.9, 62.0). There were statistically significant differences in OIDP prevalence among children in different locations (*P* < 0.05), children with or without siblings (*P* < 0.001), and children with different maternal educations (*P* < 0.001). Children with active caries were more likely to report OIDP (68.0%, 95% CI 62.9–72.7) than those without active caries (58.4%, 95% CI 56.1–60.7). The largest impact was reported on eating (41.8%, 95% CI 39.8–43.9), followed by mouth cleaning (28.5%, 95% CI 26.6–30.4) and smiling (28.5%, 95% CI 26.6–30.4), and the smallest impact was reported on studying (11.9%, 95% CI 10.6–13.3).

**Table 1:** Distribution of participants’ demographic characteristics and oral health problems; OIDP score and OIDP prevalence (OIDP score > 0) with differences in distribution and mean ranks

	n (%)	OIDP score, mean (SD)	OIDP prevalence, % (95%CI)
Total	2158	1.76 (2.11)	60.0 (57.9, 62.0)
Gender			
Male	1079 (50.0%)	1.73 (2.15)	57.5 (54.5, 60.4)
Female	1079 (50.0%)	1.79 (2.08) ^NS^	62.5 (59.5, 65.3) *
Single-child			
Yes	1575 (73.0%)	1.66 (2.10)	56.9 (54.4, 59.3)
No	583 (27.0%)	2.03 (2.13) ***	68.3 (64.4, 71.9) ***
Residence			
Urban	1643 (76.1%)	1.66 (2.11)	56.8 (54.4, 59.2)
Rural	515 (23.9%)	2.07 (2.08) ***	70.1 (66.0, 73.9) ***
Maternal education			
≤ Junior middle school	513 (23.8%)	2.11 (2.22)	69.2 (65.1, 73.0)
High school	589 (27.3%)	1.70 (1.97)	61.1 (57.1, 65.0)
College school	413 (19.1%)	1.66 (2.21)	54.5 (49.6, 59.2)
University or above	643 (29.8%)	1.54 (2.06) ***	55.1 (51.2, 58.9) ***
Active caries			
Absent	1811 (83.9%)	1.73 (2.12)	58.4 (56.1, 60.7)
Present	347 (16.1%)	1.93 (2.06) **	68.0 (62.9, 72.7) ***
Gingival bleeding			
Absent	1870 (86.7%)	1.78 (2.14)	59.9 (57.7, 62.1)
Present	288 (13.3%)	1.66 (1.97) ^NS^	60.4 (54.7, 65.9) NS
Calculus			
Absent	1202 (55.7%)	1.78 (2.13)	60.6 (57.8, 63.3)
Present	956 (44.3%)	1.73 (2.10) ^NS^	59.2 (56.1, 62.3) ^NS^

NS: Non-significance, *<0.05, **<0.01, ***<0.001

### OIDP prevalence for single and non-single children

The OIDP prevalence of non-single children (68.3%) was higher than that of single children (56.9%). Compared to single children, non-single children were more likely to report impacts on eating (*P* < 0.001), speaking (*P* = 0.009), mouth cleaning (*P* = 0.019), emotion (*P* = 0.017), and smiling (*P* = 0.001). No statistically significant differences were shown in sleeping, studying, and social contact impacts between single and non-single children (Table 2).

**Table 2:** Percentage (95% CI) of impact for 8 items on child-OIDP scale for total, single, and non-single children

	Total	Single children	Non-single children	*P* value
	(N = 2158)	(n = 1575)	(n = 583)	
Eating	41.8 (39.8, 43.9)	39.0 (36.7, 41.5)	49.4 (45.4, 53.5)	< 0.001 ***
Speaking	14.7 (13.3, 16.3)	13.4 (11.2, 15.8)	18.0 (15.1, 21.4)	0.009 **
Mouth cleaning	28.5 (26.6, 30.4)	27.1 (25.0, 29.4)	32.2 (28.6, 36.2)	0.019 *
Sleeping	13.4 (12.1, 14.9)	13.1 (11.5, 14.8)	14.4 (11.8, 17.5)	0.422 ^NS^
Emotion	21.7 (20.0, 23.5)	20.4 (18.5, 22.5)	25.2 (21.9, 28.9)	0.017 *
Smiling	28.5 (26.6, 30.4)	26.4 (24.3, 28.6)	34.0 (30.2, 37.9)	0.001 **
Study	11.9 (10.6, 13.3)	11.5 (10.0, 13.2)	12.9 (10.4, 15.8)	0.381 ^NS^
Social contact	15.5 (14.1, 17.1)	14.9 (13.2, 16.7)	17.3 (14.5, 20.6)	0.160 ^NS^

NS: Non-significance, * < 0.05, ** < 0.01, *** < 0.001

### Impacts of demographic characteristics and oral health problems on OIDP

Table 3 shows the regression results of socio-demographic characteristics and oral health problems on OIDP. Female, rural, non-single children, and children with less educated mothers were more likely to report OIDP. Children with active caries were more likely to report OIDP (OR = 1.42, *P* < 0.01), while children with bleeding or having dental calculus were not. After adjusting for other socio-demographic variables and oral health problems, the OR for non-single children was 1.31 (*P* < 0.01).

**Table 3:** Child-OIDP (0 = no impact, 1 = at least one impact) regressed on socio-demographical characteristics and oral health problems: OR and 95% CI, unadjusted and adjusted analyses

	Unadjusted OR (95%CI)	Adjusted OR (95%CI)
*Socio-demographical characteristics*		
Gender		
Male	1.00	1.00
Female	1.23 (1.04, 1.46) *	1.24 (1.04, 1.48) *
Single-child		
Yes	1.00	1.00
No	1.63 (1.33, 1.99) ***	1.31 (1.05, 1.63) *
Residence		
Urban	1.00	1
Rural	1.78 (1.44, 2.21) ***	1.43 (1.13, 1.81) **
Maternal education		
≤ Junior middle school	1.00	1.00
High school	0.70 (0.55, 0.99) **	0.81 (0.62, 1.05) ^NS^
College school	0.53 (0.41, 0.70) ***	0.67 (0.50, 0.89) **
University or above	0.55 (0.43, 0.70) ***	0.71 (0.54, 0.93) *
*Oral health problems*		
Active caries		
Absent	1.00	1.00
Present	1.53 (1.18, 1.93) **	1.42 (1.11, 1.82) **
Gingival bleeding		
Absent	1.00	1.00
Present	1.02 (0.79, 1.32) ^NS^	1.04 (0.79, 1.37) ^NS^
Calculus		
Absent	1.00	1.00
Present	0.95 (0.80, 1.12) ^NS^	0.92 (0.76, 1.11) ^NS^

NS: Non-significance, * < 0.05, ** < 0.01, *** < 0.001

### The interaction effect of siblings’ presence and location on OIDP

Additionally, an examination of the interaction effect between presence of siblings and locations on OIDP was conducted. As expected, OIDP prevalence was highest among non-single and rural children (Table 4). Compared with single and urban children, the adjusted OR for single and rural children was 1.28 (0.96, 1.71), for non-single and urban children was 1.12 (0.92, 1.56), and for non-single and rural children was 2.03 (1.47, 2.81). The synergy index between the siblings and residence was 2.18 (1.30, 3.67), indicating that there is a synergistic interaction effect on OIDP.

**Table 4:** The interaction effect between presence of siblings and locations on OIDP

	Total	OIDP,	OR	S
		n (%)	(95% CI) ^a^	(95%, 85% CI) ^a^
Single and Urban	1324	734 (55.4%)	1	2.18
Single and Rural	251	162 (64.5%)	1.28 (0.96, 1.71)	(0.72, 6.62),
Non-single and Urban	319	199 (62.4%)	1.12 (0.92, 1.56)	(1.30, 3.67)
Non-single and Rural	264	199 (75.4%)	2.03 (1.47, 2.81)	

^a^Adjusted for gender, maternal education, and oral health problems

## Discussion

This is the one of few studies assessing the OHRQoL of Chinese schoolchildren. The first contribution of this study is assessing the effect of siblings’ presence on children’s OHRQoL. Results show that non-single children were more likely to report OIDP and have lower OHRQoL. The second contribution is testing the interaction effect between siblings’ presence and locations on children’s OHRQoL. An excessive risk for OIDP was observed among non-single and rural children.

The prevalence of OIDP in Chinese schoolchildren was 60.0%, which was similar to children of the same age group in Sudan (52%) [27] and Uganda (62%) [34], but lower than those in Thailand (85.2%) [35]. The highest impact reported in the current sample was on eating (41.8%), which is consistent with previous studies [27, 35, 36].

Only active caries was negatively associated with children’s OHRQoL in this study, which were consistent with previous studies [27, 37]. Active caries cause pain, discomfort, and functional illimitation, which explains the association of active caries and OHRQoL. Previous studies showed that gingival bleeding was not associated with children’s OHRQoL [27, 37, 38], similar result was found in this study. However, a negative relationship between extensive calculus and/or gingivitis and children’s OHRQoL was found in some literatures [39]. The inconsistent results might partial due to the measurements, since many studies with non-significant results only report presence or absence of gingival bleeding (gingivitis) and lack assessment of the severity and extent of gingivitis. Thus, future studies are encouraged to apply more precise method in measuring severity [27] and extent of gingivitis [39], which helps to find a more solid evidence in association between periodontal health status and children’s OHRQoL.

Rural children or non-single children were more likely to report the lower OHRQoL in this study. Based on the social determinants of oral health, demographical factors might have indirect effects on OHRQoL via oral health status or dental care utilization [40], which could explain the association between demographical factors and OHRQoL. Previous studies show that the sibling number decreased the chance of annual dental visit and increased the chances of having caries [16, 17]. Dental visit and caries were positively and negatively associated with OHRQoL, respectively [20, 41, 42]. These series of evidences indicate that single children tend to have better oral health status as well as OHRQoL. One-child policy effectively encourage parents to have only one child leading to positive consequences for child’s physical health status [43]. Our findings support the policy-related determinants of child oral health. First, one-child policy led to greater involvement by parents in child’s care [44]. It is important for parents to participate in and supervise children’s tooth brushing in early childhood, which is highly related to oral hygiene tooth-brushing habits in later childhood. Second, one-child policy limits siblings’ presence, which can decrease parental dental neglect and increase the possibility of children’s dental care utilization [16, 45].

However, association of demographical variables and OHRQoL is relatively low. The following reasons might explain the low ORs: first, Beijing government has been conducting an oral health program for schoolchildren since 2005, including offering oral health education, oral health examinations, and pit and fissure sealing [46]. This oral public health care might narrow oral health gap among different groups since the disadvantage groups enjoy more benefits from free public health service [47]. Second, since demographical factors might have indirect effects on OHRQoL via oral health status, inclusion of the dental variables weakens the ORs on demographical factors.

In this study, the synergistic interaction results showed that the excessive risk increase for OIDP among non-single and rural children, but the effects were non-significant for non-single and urban children or for single and rural children. First, for non-single and urban children, the oral healthcare resources are more accessible and parental oral health awareness are higher in urban area, which might alleviate the negative impact of sibling’s number [48]. Second, for the single-rural children, although they were inaccessible to community’s oral healthcare, their parents were more likely to participate or invest in children’s oral health as being the only-child in the family [45]. Enjoying sufficient oral healthcare resources in family environment might reduce the negative impact of living in rural area. However, a synergistic interaction effect was found among non-single and rural children was found at the 85% confidence intervals. As the application of the confidence level (95% CI) to the interaction significance test could obscure a possible synergism, an 80% CI was applied in the previous interaction study [31]. In the current study, an 85% CI was applied. In this study, the excessive risk increase for OIDP was observed among those who were non-single and rural children. The maldistribution of oral healthcare resources between rural and urban areas might contribute to the additional risk. Oral healthcare resources were unequally distributed in urban and rural areas in China [49, 50], and one third of rural residents failed to use the oral health services because of the long distance to dental clinics [51]. Non-single and rural children are exposed to insufficient oral healthcare resources per capita in a family and have poor accessibility of oral healthcare services in the community, and these two factors combined may lead to the interaction effect. Moreover, one child policy was more strictly enforced in urban areas [52]. Overlapping of location and sibling factor might contribute to the interaction effect. 

The findings of current study support the one-policy might have positive effects in children’s OHRQoL. China unveiled the universal two‐child policy since 2016, and family size will be larger in the future [53]. To improve children's oral health and oral health equity, oral health policy makers should pay attention to the impact of family planning policy on children's oral health and the corresponding policy should be developed when necessary.

Several limitations of the current study should be mentioned. Due to the one child policy implemented in China, we only collected data on single or non-single children and failed to investigate the impacts of the number of siblings on children’s OHRQoL. It is well known that one-child policy affected the sex ratio in China [54], and sex ratio is not likely to change current result. According to statistical data of Beijing in 2015, male to female ratio among Beijing children aged 10–14 years is 107:100, which is close to 1:1. Results from the gender-weighted data were consistent with current results, which further suggests that the sex ratio is not likely to change current result. However, the national sex ratio was more imbalanced than that in Beijing. When the evidence is applied to other children, the possible impact of gender should be considered. Due to sample size limitation, we did not conduct gender-stratified analysis, but we included gender as a controlled variable in this study and tried to control its effect. Future research can further test the multiple interaction among gender, single children, and location. Besides, only the severity of oral health daily impacts was assessed, and the OIDP score and prevalence were calculated in this study. Future studies could apply the original version assessing the frequency and severity of both impacts and conduct a more detailed analysis.

## Conclusions

Children with siblings are more likely to report a lower OHRQoL, which indicates that the quantity-quality trade-off might exist in children’s OHRQoL. The synergistic interaction effect between presence of sibling and place of residence on children’s OHRQoL was found, suggesting that non-single and rural children are vulnerable population in oral health. These finding suggests that oral health intervention programs should give priority to non-single and rural children.

## Supplementary Information

**Additional file 1.** **Supplementary Table 1.** Internal reliability analysis of Child-OIDP: Items correlation matrix.

**Additional file 2.** **Supplementary Table 2.** Distribution of participants' demographic characteristics and oral health problems: Missing and non-missing data comparison. **Supplementary Table 3.** Child-OIDP regressed on socio-demographical characteristics and oral health problems: Before and after missing data imputation.

**Additional file 3.** **Supplementary Table 4.** Child-OIDP (0= no impact, 1= at least one impact) regressed on socio-demographical characteristics and oral health problems: Gender-weighted data analysis.

## Data Availability

Data used and/or analyzed during the current study are available from the corresponding author on reasonable request.

## Abbreviations

OHRQoL: Oral Health Related Quality of Life
WHO: World Health Organization
OIDP: Oral Impacts on Daily Performance
SC: Simple Count
DT: Decayed Teeth
CI: Confidence interval
OR: Odds ratio
SI: Synergy Index
NS: Non-significance
