# Supplementary material for: Association of siblings’ presence and oral health-related quality of life among children: a cross-sectional study
Source: BMC Oral Health. 2021 Mar 24;21:153. doi: 10.1186/s12903-021-01526-y (PMC7988954; doi:10.1186/s12903-021-01526-y)
Supplement: Supplementary file 1 — Additional file 1. Supplementary Table 1. Internal reliability analysis of Child-OIDP: Items correlation matrix. [file 12903_2021_1526_MOESM1_ESM.docx]

| Supplementary table 1. Internal reliability analysis of Child-OIDP: Items correlation matrix. | | | | | | | | | |
| --- | --- | --- | --- | --- | --- | --- | --- | --- | --- |
|  | Eating | Speaking | Mouth cleaning | Sleeping | Emotion | Smiling | Study | Social contact | Alpha if item deleted |
| Eating | 1.00 |  |  |  |  |  |  |  | 0.839 |
| Speaking | 0.45 | 1.00 |  |  |  |  |  |  | 0.841 |
| Cleaning mouth | 0.55 | 0.38 | 1.00 |  |  |  |  |  | 0.842 |
| Sleeping | 0.42 | 0.41 | 0.43 | 1.00 |  |  |  |  | 0.835 |
| Emotion | 0.42 | 0.36 | 0.37 | 0.44 | 1.00 |  |  |  | 0.831 |
| Smiling | 0.34 | 0.37 | 0.34 | 0.43 | 0.58 | 1.00 |  |  | 0.837 |
| Study | 0.42 | 0.47 | 0.41 | 0.61 | 0.47 | 0.42 | 1.00 |  | 0.834 |
| Social contact | 0.36 | 0.42 | 0.36 | 0.43 | 0.57 | 0.58 | 0.47 | 1.00 | 0.833 |
