# Supplementary material for: Association of siblings’ presence and oral health-related quality of life among children: a cross-sectional study
Source: BMC Oral Health. 2021 Mar 24;21:153. doi: 10.1186/s12903-021-01526-y (PMC7988954; doi:10.1186/s12903-021-01526-y)
Supplement: Supplementary file 2 — Additional file 2. Supplementary Table 2. Distribution of participants' demographic characteristics and oral health problems: Missing and non-missing data comparison. Supplementary Table 3. Child-OIDP regressed on socio-demographical characteristics and oral health problems: Before and after missing data imputation. [file 12903_2021_1526_MOESM2_ESM.docx]

| Supplementary Table 2. Distribution of participants' demographic characteristics and oral health problems: Missing and non-missing data comparison | | | |
| --- | --- | --- | --- |
|  | Non-missing data  (n = 1868) | Missing data  (n = 290) ^a^ | *P* -value |
| **Gender** |  |  |  |
| Male | 933 (49.9%) | 146 (50.3%) | 0.900 |
| Female | 935 (50.1%) | 144 (49.7%) |  |
| **Single-child** |  |  |  |
| Yes | 1360(72.8%) | 215 (74.1%) | 0.634 |
| No | 508 (27.2%) | 75(25.9%) |  |
| **Rural-urban status** |  |  |  |
| Urban | 1413 (75.6%) | 230 (79.3%) | 0.173 |
| Rural | 455 (24.4%) | 60 (20.7%) |  |
| **Active caries** |  |  |  |
| Absence | 1564 (83.7%) | 247 (85.2%) | 0.533 |
| Present | 304 (16.3%) | 43 (14.8%) |  |
| **Gingival bleeding** |  |  |  |
| Absence | 1608 (86.1%) | 262 (90.3%) | 0.047 |
| Present | 260 (13.9%) | 28 (9.7%) |  |
| **Calculus** |  |  |  |
| Absence | 1045 (55.9%) | 157 (54.1%) | 0.565 |
| Present | 823 (44.1%) | 133 (45.9%) |  |
| **OIDP** |  |  |  |
| Absence | 746 (39.9%) | 118 (40.7%) | 0.807 |
| Present | 1868 (60.1%) | 172 (59.3%) |  |
| a: There were 290 missing data in maternal education. | | | |

| Supplementary Table 3. Child-OIDP regressed on socio-demographical characteristics and oral health problems: before and after missing data imputation | | |
| --- | --- | --- |
|  | Before data imputation (N=1868) | After data imputation (N=2158) |
| **Socio-demographical characteristics** | | |
| Gender |  |  |
| Male | 1.00 | 1.00 |
| Female | 1.27 (1.05, 1.54) * | 1.24 (1.04, 1.48) * |
| Single-child |  |  |
| Yes | 1.00 | 1.00 |
| No | 1.34 (1.05, 1.71) * | 1.31 (1.05, 1.63) * |
| Residence |  |  |
| Urban | 1.00 | 1.00 |
| Rural | 1.39 (1.08, 2.78) * | 1.43 (1.13, 1.81) ** |
| Maternal education | |  |
| ≤ Junior middle school | 1.00 | 1.00 |
| High school | 0.79 (0.60, 1.03) NS | 0.81 (0.62, 1.05) ^NS^ |
| College school | 0.65 (0.47, 0.88) ** | 0.67 (0.50, 0.89) ** |
| University or above | 0.67 (0.50, 0.90) ** | 0.71 (0.54, 0.93) * |
| **Oral health problems** | |  |
| Active caries |  |  |
| Absence | 1.00 | 1.00 |
| Presence | 1.48 (1.13, 1.93) ** | 1.42 (1.11, 1.82) ** |
| Gingival bleeding |  |  |
| Absence | 1.00 | 1.00 |
| Presence | 1.01 (0.76, 1.36) ^NS^ | 1.04 (0.79, 1.37) ^NS^ |
| Calculus | |  |
| Absence | 1.00 | 1.00 |
| Presence | 0.97 (0.79, 1.18) ^NS^ | 0.92 (0.76, 1.11) ^NS^ |
| NS: Non-significance, *<0.05, **<0.01, ***<0.001. | | |
