# Supplementary material for: Association of siblings’ presence and oral health-related quality of life among children: a cross-sectional study
Source: BMC Oral Health. 2021 Mar 24;21:153. doi: 10.1186/s12903-021-01526-y (PMC7988954; doi:10.1186/s12903-021-01526-y)
Supplement: Supplementary file 3 — Additional file 3. Supplementary Table 4. Child-OIDP (0= no impact, 1= at least one impact) regressed on socio-demographical characteristics and oral health problems: Gender-weighted data analysis. [file 12903_2021_1526_MOESM3_ESM.docx]

| Supplementary Table 4. Child-OIDP (0= no impact, 1= at least one impact) regressed on socio-demographical characteristics and oral health problems: Gender-weighted data analysis | | |
| --- | --- | --- |
|  | Unadjusted OR (95%CI) | Adjusted OR (95%CI) |
| Socio-demographical characteristics | |  |
| Gender |  |  |
| Male | 1.00 | 1.00 |
| Female | 1.23 (1.04, 1.46) * | 1.24 (1.04, 1.48) * |
| Single-child |  |  |
| Yes | 1.00 | 1.00 |
| No | 1.64 (1.33, 2.01) *** | 1.31 (1.06, 1.64) * |
| Residence |  |  |
| Urban | 1.00 | 1.00 |
| Rural | 1.79 (1.50, 2.22) *** | 1.43 (1.13, 1.81) ** |
| Maternal education |  |  |
| ≤Junior middle school | 1.00 | 1.00 |
| High school | 0.70 (0.54, 0.90) ** | 0.81 (0.62, 1.05) ^NS^ |
| College school | 0.53 (0.41, 0.70) *** | 0.67 (0.50, 0.89) ** |
| University or above | 0.54 (0.43, 0.69) *** | 0.71 (0.54, 0.93) * |
| Oral health problems |  |  |
| Active caries |  |  |
| Absence | 1.00 | 1.00 |
| Presence | 1.52 (1.19, 1.94) ** | 1.42 (1.11, 1.83) ** |
| Gingival bleeding |  |  |
| Absence | 1.00 | 1.00 |
| Presence | 1.01 (0.79, 1.30) | 1.03 (0.79, 1.36) ^NS^ |
| Calculus |  |  |
| Absence | 1.00 | 1.00 |
| Presence | 0.94 (0.79, 1.12) | 0.91 (0.76, 1.10) ^NS^ |
| NS: Non-significance, *<0.05, **<0.01, ***<0.001. | | |

| Supplementary Table 5. The interaction effect between presence of siblings and locations on OIDP: Gender-weighted data analysis | | | |
| --- | --- | --- | --- |
|  |  | OR, (95% CI) ^a^ | S, (95%, 85% CI) a |
| Single | Urban | 1.00 | 2.15 |
|  | Rural | 1.28 (0.96, 1.72) | (0.72, 6.43), |
| Non-single | Urban | 1.20 (0.93, 1.57) | (1.27, 3.66) |
|  | Rural | 2.05 (1.48, 2.84) |  |
| a: adjusted for gender, maternal education, and oral health problems. | | | |
